# Understanding the value of a doctorate for allied health professionals in practice in the UK: a survey

**DOI:** 10.1186/s12913-024-11035-7

**Published:** 2024-05-02

**Authors:** Jo Watson, Steven Robertson, Tony Ryan, Emily Wood, Jo Cooke, Susan Hampshaw, Hazel Roddam

**Affiliations:** 1 Dr Jo Watson Consulting Ltd., Hampshire, UK; 2 Division of Nursing and Midwifery, Health Sciences School, University of Sheffield, Sheffield, UK; 3 School of Health and Related Research, Health Sciences School, University of Sheffield, Sheffield, UK; 4 NIHR Health Determinants Research Collaboration, Doncaster, UK; 5 Health Education England, Manchester, UK

**Keywords:** Allied health professions, Doctorate, Value, Research capacity, Research capability building, Research culture, Service improvement, Workforce development

## Abstract

**Background:**

The need to transform the United Kingdom’s (UK) delivery of health and care services to better meet population needs and expectations is well-established, as is the critical importance of research and innovation to drive those transformations. Allied health professionals (AHPs) represent a significant proportion of the healthcare workforce. Developing and expanding their skills and capabilities is fundamental to delivering new ways of working. However, career opportunities combining research and practice remain limited. This study explored the perceived utility and value of a doctorate to post-doctoral AHPs and how they experience bringing their research-related capabilities into practice environments.

**Methods:**

With a broadly interpretivist design, a qualitatively oriented cross-sectional survey, with closed and open questions, was developed to enable frequency reporting while focusing on the significance and meaning participants attributed to the topic. Participants were recruited via professional networks and communities of practice. Descriptive statistics were used to analyse closed question responses, while combined framework and thematic analysis was applied to open question responses.

**Results:**

Responses were received from 71 post-doctoral AHPs located across all four UK nations. Findings are discussed under four primary themes of utilisation of the doctorate; value of the doctorate; impact on career, and impact on self and support. Reference is also made at appropriate points to descriptive statistics summarising closed question responses.

**Conclusion:**

The findings clearly articulate variability of experiences amongst post-doctoral AHPs. Some were able to influence team and organisational research cultures, support the development of others and drive service improvement. The challenges, barriers and obstacles encountered by others reflect those that have been acknowledged for many years. Acknowledging them is important, but the conversation must move forward and generate positive action to ensure greater consistency in harnessing the benefits and value-added these practitioners bring. If system-wide transformation is the aim, it is inefficient to leave navigating challenges to individual creativity and tenacity or forward-thinking leaders and organisations. There is an urgent need for system-wide responses to more effectively, consistently and equitably enable career pathways combining research and practice for what is a substantial proportion of the UK healthcare workforce.

**Supplementary Information:**

The online version contains supplementary material available at 10.1186/s12913-024-11035-7.

## Background

The imperative to transform the delivery of health and social care in the United Kingdom (UK) to better meet the changing needs and expectations of the population has been acknowledged for some time. Equally well recognised is the critical importance of research and innovation to drive those transformations, including advances in treatments and interventions [1, 2]. As the third largest clinical workforce in the UK’s National Health Service (NHS), the allied health professions^1^ (AHPs) are acknowledged as having an essential role in helping meet demand. In addition, their impact reaches beyond the NHS with significant contributions made across the health and care sector in roles within social care, housing, early years, schools, public health, the criminal justice system and in private, voluntary, community and social enterprise organisations [3].

With a fundamental need to identify new ways of working and delivering care, developing the skills and expanding the capabilities of the workforce, and creating meaningful career pathways to support retention of experienced staff, are paramount [1, 2, 4–6]. Building research capability and capacity to complement practice expertise is key to optimising the workforce and strengthening the evidence-base informing safe, clinically effective, cost efficient services [7–10]. A complex interplay between developing strong internal organisational infrastructure and supporting individual career planning and skills development has been identified [11]. Where this is successfully navigated, healthcare organisations that are research-active are noted to have improved performance and patient experience, and better staff recruitment and retention, compared to those with lower levels of research engagement [12–15].

Clinical academics are an important strand of the workforce who work concurrently in practice and academic environments and are research-active [9]. Newington et al’s [16] systematic review identified a wide range of positive impacts from non-medical clinical academics in the UK. These included benefits to patients, service provision and the workforce, including: recruitment and retention; the research profile, culture and capacity of organisations; knowledge exchange and the economy. Clinical academics themselves were also noted to benefit through, for example, increased job satisfaction, growing their networks and influence, developing leadership as well as research skills, and unlocking new career opportunities [16].

Acknowledging these potential benefits, Comer et al. [17] explored the perceived level of research capacity and culture within AHPs working in the NHS, using the Research Capacity and Culture (RCC) tool. Only 34% of respondents reported research-related activities being part of their roles, and of these, 79% had less than 25% of their time allocated for research-related activity. Further, only 18% reported that research engagement was routinely discussed at annual appraisals. Similarly, and using the same RCC tool, Cordrey et al. [18] found that 31% of responding AHPs from a single NHS department reported research-related activities as a component of their role, and of these 21% had dedicated time for research. Lack of time and opportunity are noted to curtail research engagement to a greater extent than limits in capability or ambition [17, 18].

Despite faring better than their nursing and midwifery colleagues in securing funding via National Institute for Health and Care Research (NIHR) developmental pathways [19], opportunities for AHPs (amongst others) to develop clinical academic careers remain limited [20, 21]. Further, key barriers to research engagement persist, despite having been highlighted over a number of years (see, for example [7, 22, 23]), . Even where funding has been secured, backfill to enable release from practice duties is a particular challenge [16, 18]. A related obstacle is a lack of time for research [17, 18]. This in turn is linked to the need to accommodate practice-facing and research components of roles [16], with practice roles frequently taking priority [17, 18]. It has also been noted that feelings of personal guilt can direct AHPs’ own prioritisation towards clinical workload management at the expense of engaging in research activities and their own career development [18]. The scarcity of research-engaged organisational cultures and clinical academic roles to aspire to [16] remains a foundational issue that must be addressed.

In this context, the publication of the Allied Health Professions’ Research and Innovation Strategy for England [24] was driven by recognition of the urgent need for transformational change in the pace of growth, stability and sustainability of research engagement by AHPs. Understanding more about how AHPs who have undertaken doctoral studies experience bringing their research-related capabilities into practice environments provides helpful insights to inform the actions required to progress this agenda and realise the visions outlined in the HEE Research and Innovation Strategy.

## Methods

### Study aim

To understand the perceived utility and value of a doctorate to post-doctoral Allied Health Professionals in practice in the UK.

### Study design

The overall design of this study was broadly interpretivist in nature, an approach concerned with discovering the meaning people attach to experiences and how this influences their actions [25]. A cross-sectional survey, with closed and open questions, was developed to report the frequency of participant responses and to facilitate a focus on the significance participants attributed to the research topic. In this sense, the research project and survey tool were qualitative in their orientation [26]. Mixed surveys with a qualitative emphasis (and even qualitative questionnaires) are increasingly being used in health and social care research as they limit the number of constraining responses and allow participants to provide as much information as they choose in their own terms [26].

Ethics approval was obtained from the University of Sheffield ethics committee (Ref: 023667). The Standards for Reporting Qualitative Research (SRQR) checklist for cross-sectional studies was used to guide this study’s conduct and reporting [27].

### Participants and recruitment

Nurses, midwives, healthcare scientists and AHPs currently undertaking or having completed doctorates were recruited via professional networks and via the Yorkshire and Humber Collaboration and Leadership for Allied Health and Care Research (CLAHRC) infrastructure. A Twitter© account was established for the purpose of the study and a link to an online survey was disseminated via this Twitter© feed. Within Twitter©, relevant communities of practice were targeted and encouraged to actively retweet the survey. Respondents were also asked to retweet and share the survey link within their networks to generate a diverse sample. Only those respondents based within the UK were included in the study.

### Data collection

A bespoke, 23-item questionnaire containing closed and open questions, was developed to collect information about demographics and the self-reported benefits and impact of doctoral study. Closed questions included information about: motivation; mode, length and funding sources of doctoral study; prior research experience; perceived benefits, utility and value of the doctorate; and impact on career and self. The survey also included open questions for respondents to provide greater detail about their experiences and views (see Table 1). These closed and open questions were developed by drawing on issues raised within the literature [28–30]. As is recommended in questionnaire development [31], these questions were piloted with those sharing similar characteristics to the intended survey recipients, in this case members of the Addressing Organisational Capacity to do Research Network (ACORN) community of practice. ACORN was developed as part of a capacity building programme within the Yorkshire and Humber CLAHRC. The online survey, with associated information sheet, was open from 5th Feb 2019 to 15th March 2019. Participation was optional and anonymous.

**Table 1:** Open questions

“To what extent are you able to utilise these benefits in your current role?”
“Please describe how these [doctoral] skills are valued or not by your employer”
“Could you tell us a little about your career pathway. In particular, we’d like you to reflect on how you feel your doctorate has (or hasn’t) impacted on this career”
*“Please add any other comments about your doctoral studies”*

### Analysis

For the purpose of this paper, data from AHPs was separated from the nurses/midwives and healthcare scientists. The intention is to focus on AHP experiences to complement previously published work from this dataset that focused on the reported experiences of nurses and midwives [21].

Analysis of AHP responses to closed questions, supported by IBM SPSS© software, was undertaken with descriptive statistics reported here. As noted by others [26], open question data in mixed surveys can be analysed in a thoroughly qualitative way. Open question responses from the AHPs were therefore analysed using a combined framework and thematic analysis. First stage analysis involved placing open response data into a three-theme framework developed following the earlier analysis of the data for the nurses and midwives [21]. This was conducted by SR and was cross-checked for accuracy by TR. Second stage analysis involved categorising and merging data within these themes into sub-themes. This was conducted by SR and was cross-checked for accuracy by TR. The final stage involved clustering the sub-themes into existing or new themes. This was conducted by SR, cross-checked for accuracy by TR and agreed by all research team members. The themes developed through and during the analysis differed little from the previous analysis of the data from nurses and midwives, although the sub-themes altered slightly and one previous theme was split into two themes, resulting in four themes with 13 sub-themes (see Table 2).

**Table 2:** Themes and sub-themes

Theme	Sub-Themes
Utilisation of the doctorate	Use of skills Mixed use/non-use of skills
Value of the doctorate	Doctorate valued Doctorate not valued Mixed valued/not valued Financially valued/not valued
Impact on career	Positive impact No impact/restricted opportunities A crooked path
Impact on self and support	Positive impact Negative aspects Mixed impact Support (having/not having)

Apart from the context section below on ‘route to doctoral completion’, the closed and open question data analysis is integrated and presented under the four theme headings that were derived from the analysis of the open question data.

## Results

There were 214 respondents from across the UK. They included 47 nurses, 96 healthcare scientists and 71 AHPs. Representing the AHP disciplines, respondents were: physiotherapists (PT, *n* = 26), speech and language therapists (SLT, *n* = 15), occupational therapists (OT, *n* = 9), radiographers (RAD, *n* = 8), podiatrists (POD, *n* = 4), dieticians (DIET, *n* = 3), art therapists (*n* = 2), paramedics (*n* = 2), a music therapist (*n* = 1), and an orthoptist (*n* = 1). To aid anonymity, art therapists, paramedics, music therapists and orthoptists were all coded as MISC. Table 3 summarised the geographical spread of this self-selecting sample. As noted earlier, only data from the AHP survey respondents are reported here.

**Table 3:** Geographical spread of respondents

Country	Region	Number
Scotland	-	7
Wales	-	2
Northern Ireland	-	1
England	North East	6
	North West	3
	Yorkshire and Humber	11
	East Midlands	2
	West Midlands	3
	East of England	1
	London	17
	South East	8
	South West	10
**TOTAL**	-	**71**

### Route to doctoral completion

All AHP respondents had completed study at doctoral level; none were still undertaking their studies. One-third had studied full-time (30%, *n* = 21). Well over half of AHP respondents (56%, *n* = 40) had taken five years or fewer to complete, with 29 (41%) taking more than five years (2 missing). Just under half of AHP respondents had some form of research experience prior to commencing their doctorates (46%, *n* = 33). These experiences were typically formed through internships (including NIHR opportunities), Master’s dissertation study, secondments and co-investigator roles.

### Post-doctoral experiences

The following section outlines the findings from the analysis of the open question data which allowed respondents to reflect upon their current professional experiences in a post-doctoral context. However, at appropriate points references are also made to some of the descriptive statistics summarising responses to closed question items included within the questionnaire. These data are discussed under four theme headings (see Table 2) with illustrative data quotations.

### Utilisation of the doctorate

Most participants who commented mentioned utilising the skills and knowledge gained through their doctoral studies. For some, this related directly to aspects of clinical care including shifting team culture and changing existing practices:

*“I now influence my team’s way of thinking about what we do with patients. We are all more analytical and confident to question practices that have been historically used for many years.” [PT4]*.

*“My research and critical thinking contributes to redesigned pathways and patient outcome improvement” [RAD6]*.

Important here was the recognition that developing, enhancing or extending the skill of critical thinking was a key aspect that helped drive these service improvements and change:

*“I think my critical analysis skills and research skills have helped significantly in developing our service and providing effective evidence-based interventions for the patients seen by our team […] My doctorate has given me many transferable skills and has enabled me to deliver much better evidence-based care for my clients and our service.” [SLT14]*.

As well directly linking to service improvement and change, the doctoral journey was noted to enhance a wider skill-base that is transferrable and applicable across roles, and across teams, particularly, but not solely, in relation to the research aspects of their practice:

*“The doctorate allowed me to deepen my research and clinical expertise somewhat but the benefits in terms of ‘soft-skills’; e.g. time management, resilience, negotiation have been enormous.” [RAD3]*.

*“These skills have filtered through to my clinical work, helped me to facilitate service changes within the clinical team and helped to foster an ethos of research as core business within my immediate team, but also more widely in the hospital Trust and wider professional networks.” [SLT13]*.

A further skill developed by some through the doctoral journey was an improved ability (and confidence) to train, educate, supervise and encourage others:

*“I utilise these benefits on a daily basis […] I teach and train colleagues where I work to be able to critically appraise research and consider the applications for clinical practice. I also lecture and believe that my experience clinically and with research is an important combination.” [SLT12]*.

While the majority of comments were positive, there was also suggestion that the ability to utilise the skills learnt through doctoral experiences was not always present. Almost one-third agreed or strongly agreed with the statement: *“there are limited opportunities to use skills gained during my doctorate”* (31%, n = 22). One-quarter of participants felt over-qualified for their role (24%, n = 18). There were some general comments in response to the question *“To what extent are you able to utilise these benefits in your current role?”*, such as; *“Limited” [POD3]*, *“Limited ability” [DIET2]*, and *“To some degree” [DIET1]*. Others, expanding on this, demonstrated some frustration with the opportunities available to utilise the skills and knowledge developed through their doctoral studies, particularly in clinical contexts:

*“Research sits in an uncertain position in my organisation so doctorate level skills are difficult to show the benefit of.” [MISC7]*.

*“Now I’m in an academic role, completely […] In a university department I could not do my job without a PhD. In the NHS they didn’t quite know how to best exploit my new skills and knowledge (or what they were).” [SLT9]*.

*“The doctorate seems to open up more opportunities outside of the NHS as opposed to within it…” [MISC7]*.

Importantly, some have worked hard to create opportunities to put the skills and knowledge learnt into practice, but this has taken a personal toll (see also later section “Impact on self”):

*“My clinical caseload is heavier now than prior to my Doctorate and I have no protected research time. Implementing and sharing my skills has taken a monumental amount of work and perseverance.” [OT8]*.

While almost all participants recognised the importance of the skills and knowledge gained from their doctoral studies, it was not always easy to put these into practice, particularly in clinical contexts. Some suggested this difficulty links to the extent to which doctorates (and research skills and knowledge generally) are valued within their organisations or profession.

### Value of the doctorate

On completion of their doctorate, almost 60% (*n* = 43) of AHPs were confident that the qualification was valued by employers, with 11% (*n* = 8) certain that their doctorate was not valued and one quarter remaining unsure (26%, *n* = 20). For many of those whose employers recognised the value doctoral learning and experience brought, this was often linked to either the importance of improved clinical expertise or to increased credibility and prestige for the individual, their team or their organisation:

*“Adds to credibility in a national role and multi-professional arena. Clinical expertise and insight adds value to strategic NHS planning associated research and development activity.” [RAD1]*.

*“My PhD brought/brings credibility not only to me but to this specialist centre.” [SLT12]*.

For those who did not think their employers valued doctoral learning and experience, or who were not sure if it was valued, comments indicate a disinterest in doctoral study – its associated skills and what they might bring - among colleagues and managers, particularly in clinical settings:

*“My NHS role respect my clinical leadership but not my research leadership as much. It’s not considered core business.” [POD1]*.

*“Having a PhD was not valued in my previous job in the NHS because I was seen as developing skills in the wrong area - extremely disappointing for me.” [OT7]*.

*“My employer is oblivious to them [doctoral skills gained].[…] My NHS employer has no interest in my academic skills, experience or knowledge. No-one at work acknowledges my doctorate, uses Dr when addressing me or writing to me, or recognises in a positive sense the study I have undertaken.” [PT24]*.

A more frequent response to the question about whether employers valued doctoral learning and experience was to present a nuanced view of how, and by whom, the doctoral skills and knowledge were valued. For those with combined academic and clinical roles, it was often stated that the doctorate was valued in the academic context but not in their clinical role:

*“I have two roles - these skills are valued in my academic job. Maybe less so in my clinical job.” [MISC1]*.

For those employed fully within a clinical setting, important differences were noted regarding who valued what doctoral study could bring and which elements were valued, although this wasn’t consistent:

*“Locally (departmental) I think they are valued. On a hospital basis, I am not sure.” [RAD2]*.

*“I think they are respected by senior colleagues but I find my own departmental managers find little value in either higher education achievement or research, which they often consider to be a burden.” [PT3]*.

Finally, participants noted whether their doctoral studies were valued in financial terms. Around one-third of AHPs regarded their current earnings to be misaligned with their post-doctoral expectations, with 27% (*n* = 19) agreeing or strongly agreeing with the statement: *“My post-doctoral earnings are lower than I envisaged”*. The following comments were a little contradictory but are suggestive of a negative, or at best static, influence on post-doctoral earning capacity:

*“I feel it’s enhanced my career clinically and my national leadership and teaching profile, but not my earning potential.” [POD1]*.

*“PhD has been very rewarding intellectually and clinically. However, it’s offered less job security and absolutely no financial rewards, my grading remaining static since pre PhD.” [SLT7]*.

*“I wouldn’t have my lecturer job without it. Although ironically I now work at a lower clinical band so that I can maintain a clinical foothold.” [PT17]*. 

This issue of progression and financial value links to larger issues of the impact on careers that participants experienced following completion of their doctoral study.

### Impact on career

A large proportion of respondents reported being in a clinical academic post at the time of the survey (41%, *n* = 29), whilst just under one-third (28%, *n* = 20) were in academic positions. A small proportion remained in clinical positions (11%, *n* = 8), with the remainder being in what were described as managerial and leadership roles (20%, *n* = 14).

Many participants noted the positive impact that completing doctoral studies had on their career. For several, completing a doctorate facilitated or cemented an academic career path:

*“I started my PhD whilst in clinical practice and during my studies I took a role in academia. It was pivotal in being offered a position at a university where a doctorate, or working towards one, is required.” [OT2]*.

Such positive comments were also made in relation to clinical and clinical academic career development:

*“As a reporting radiographer, my role was a blend of image reporting and acquisition. My PhD facilitated growth and progression to a consultant clinical academic position.” [RAD6]*.

*“Career, research and collaborative opportunities arise much more for me post-PhD compared with pre-PhD. I seem to have greater credibility. I have freedom to choose what I do with my career now.” [PT11]*.

However, not all participants experienced this positive career impact. Some felt that undertaking doctoral study had limited impact, or even represented a backward step, particularly in relation to clinical career components:

*“After my PhD I had a phase of feeling it had derailed my career. I enjoyed my doctoral studies but never wanted an academic career. I felt as though I had stepped off the career ladder and struggled to get back on it.” [PT1]*.

Others experienced frustration with the limited opportunities for on-going research and concomitant post-doctoral career development:

*“I hoped that it would have enabled me to actively pursue further clinical research. However, opportunities were limited when I moved to [geographical area] […] I ended up falling into a management post and find it difficult now to downgrade / get opportunity to be involved directly in research.” [DIET2]*.

*“I had high hopes that this role would provide networking and research opportunities as it’s within a large teaching hospital. Despite trying to develop an AHP research culture, there is no dedicated time or support for this to happen. […] I am desperate to progress but can’t seem to navigate into that split clinical academic world that seems to be made for medics only.” [SLT11]*.

For many participants it was not a clear-cut case of whether completing doctoral level study had or had not impacted their career. Rather, most described a somewhat crooked path of post-doctoral career development; a mixture of opportunities and barriers:

*“I had diverse skills that didn’t necessarily follow a recognised path. Pleased to say that things are falling into place and after a couple of stepping stones I am finding roles that value diverse experience and are an appropriate grade/salary. The PhD helped me get here but it wasn’t a straightforward path.” [PT1]*.

*“I loved doing my PhD. However there is no career pathway for me to follow. I was lucky to be employed in a research management role and have been lucky to gain funding to continue with my research career.” [RAD2]*.

*“I feel my doctorate has given me a platform to carry out more research; however I wasn’t expecting on having to leave my senior leadership position in the NHS to do this.” [OT7]*.

What becomes clear from the above is that it often took a significant amount of personal effort, resilience and flexibility to generate a positive post-doctoral career path. This can take its toll on individual AHPs and those around them.

### Impact on self and support

There was overwhelming recognition that the doctoral experience led to changes in relation to skills and resourcefulness. Evidence amongst respondents suggested that the doctoral experience facilitated positive changes in relation to: critical thinking (100%, *n* = 71), research skills (100%, *n* = 71), specialist knowledge (97%, *n* = 69), fresh perspective (90%, *n* = 64), resilience and confidence (83%, *n* = 59) and problem solving (93%, *n* = 66).

Some participants provided positive personal accounts of undertaking doctoral study and the impact it had for them in terms of satisfaction, confidence and resilience:

*“Deciding to undertake my doctorate part-time and remain part-time in clinical practice was the best decision I made.” [POD2]*.

*“I feel that my doctoral experience has changed the way I think about everything and I continue to be thirsty for further research. I love this feeling! […] I loved it, and would always recommend others to do so for their own benefit, even if it won’t benefit their career.” [RAD5]*.

For a few, however, it represented a difficult journey having a negative impact through increased stress, the exertion of considerable effort for little gain, and disruptions to career and family:

*“It’s [PhD] hard, requires perseverance and tenacity and no guarantees of anything at the end!” [MISC3]*.

*“Being a clinical academic is problematic when it comes to stability in posts, equality in promotion, etc. Pursuing this career has resulted in many challenges in gaining recognition, promotion, work-life balance, etc.” [PT7]*.

*“I would have liked to have had a clinical-research career, but there is no support for this, it’s something I would have to carve out myself, and due to other pressures (family, financial, etc.), I just haven’t felt able to do this.” [SLT1]*.

The majority presented a mixed picture of the personal cost and impact, describing both the difficulties and the benefits doctoral study brought and the personal change it produced:

*“I have enjoyed the journey immensely and feel it was the right pathway for me. That said, it is tough and maintaining a career in research as an AHP requires not only resilience and perseverance but a willingness and ability to take risks. Job security is still uncertain and as the main breadwinner that is a big concern.” [SLT2]*.

*“It was the hardest challenge of my life. I’m still recovering and re-orientating as I changed so much during my registration period. Colleagues in clinical settings often don’t appreciate the internal changes a PhD brings which can be frustrating. It’s also not great for work/life balance at all… tough on mental health at times too. I’d absolutely do it again though because of the value it has brought me personally.” [POD4]*.

Support, including that from family, was clearly important in facilitating positive personal experiences of doctoral study and for positive post-doctoral experiences. Sources of financial support to undertake a doctorate were varied. The most cited form of support was self-funding (25%, *n* = 18), typically alongside the use of smaller funds (such as regional HEE, charity and continuing professional development funds) used during study programmes. Employer support (17%, *n* = 12) and charitable trusts were also highly cited (23%, *n* = 17). NIHR funding (including that from Fellowships and CLAHRCs) supported 13 (18%) respondents and higher education in was also cited as a significant source of financial support (12%, *n* = 9) (missing *n* = 2). This reliance upon self-funding may have contributed to almost two-fifths of respondents agreeing or strongly agreeing with the statement: *“My doctoral study was a financial risk”*.

Beyond family and financial support, employer and colleague support in terms of allowing space and time for study, and in facilitating appropriate research and personal development opportunities, was key:

*“My employer supports my development as a clinical academic by allowing me to build research into my new role, supported by ongoing application for research funding to pay for the research portion of my post.” [SLT13]*.

*“My manager has also initiated discussions about optimising the research (and training) skills I have in terms of a new role.” [MISC8]*.

However, such support (as noted in earlier sections) was not always forthcoming in relation to on-going post-doctoral research opportunities, which could be very disheartening:

*“I felt well-supported to complete the doctorate itself but I had zero post-PhD career support, including during my first post-doc position. I think this is a real gap for AHPs doing a PhD.” [MISC6]*.

*“There is a lot of help for clinicians who wants to get into research but there is not much for researchers who need support to return to the clinical practice.” [PT15]*.

*“I would like to be a clinical academic but this is not a role valued by my Trust or managers. I have had some support from previous managers to use my research skills within my current post, but research is to some degree viewed as a luxury and clinical risk and managerial issues always take priority.” [SLT14]*.

These personal accounts of the impact of doctoral experiences on individual participants, and the potential ripple effects of that for departments and organisations, have rarely been explored in previous research. Findings here therefore comprise an original contribution to understanding the lived experience of AHP doctoral study and the pursuit of career pathways combining research and practice.

## Discussion

### Organisational benefits

Some participants in this study clearly identified the organisational benefits derived from their completion of doctoral level study. Noteworthy is their articulation of ‘value added’ across all four pillars of practice (namely: professional practice; facilitation of learning; leadership; and evidence, research and development), not solely the research pillar. Echoing the findings of Newington et al. [16] and the reflections of Cooper at al [20], the findings of this study indicate the strong potential for post-doctoral practitioners to actively contribute to, and lead, service improvements, delivery of evidence-based interventions, local workforce development and the building of team and organisational cultures of research engagement. The findings also clearly illustrate the variable nature of departmental and organisational cultures related to research. It is evident that the extent to which research is embraced and embedded as fundamental to the core business of health and care providers has a strong bearing on the extent to which organisations are able to realise the benefits of, and value added by, post-doctoral practitioners.

Where research is valued, and where organisation, service leaders and managers are willing to make the sometimes initially challenging decisions to create time and space to enable research-active practitioners, there is evidence of value to people accessing services, services, departments and organisations themselves [7, 12–16, 32]. The findings from this study highlight that some organisations / departments do very well when it comes to supporting research capacity building and engagement amongst practitioners, and reaping the associated benefits. Some are on a positive journey towards developing and embedding research within practice. While it may be perceived that other organisations remain ambivalent, apparent inaction is possibly more likely associated with a determined focus on meeting the demands placed on pressurised services. This, coupled with prioritising non research-related key performance indicators linked to service commissioning, creates a challenging backdrop against which to find the time or a way to embed research engagement into service delivery.

### National policy imperatives

Notwithstanding the genuine pressures felt by services and organisations, delays in building cultures of research engagement slow and hamper the collective progress required to respond to national policy imperatives. The CQC standards for Well Led Research in NHS Trusts, introduced in 2018, specifically require evidence that research is supported across the breadth of all services [33]. The NHS Long Term Plan [1] is a recent illustration, but is by no means the first to emphasise the role of, and need for, practice-based research engagement. Further, as our findings illustrate, organisational failure to enable practice-based research engagement becomes a contributing factor in the attrition of experienced and sometimes senior practitioners from service delivery. The findings from this sample exemplify decisions to move fully into academia, as it presents an environment where post-doctoral knowledge and skills are overtly valued. The apparent reluctance or regret expressed by some who have decided to do so is particularly telling.

The NHS People Plan [4] emphasises the need to make effective use of the full range of staff skills and experience to deliver the best possible care. It also contains a significant theme related to staff retention, identifying that ‘systems and employers must make greater efforts to design and offer more varied roles to retain our people’ (p46). Employers, line managers and supervisors are called on to ‘create the time and space for training and development … with a renewed emphasis on the importance of flexible skills and building capabilities rather than staying within traditionally-defined roles’ (p36). The findings presented here suggest that there is still some way to go to consistently implementing these approaches for those practitioners with post-doctoral careers.

This study’s findings also identify that it can be difficult for post-doctoral AHPs to find a viable pathway to return to practice, whether entirely or in clinical academic roles. Those who remain in practice, often experienced relentless barriers and obstacles to deploying their hard-won knowledge and skills. Many ended up settling for the status quo. This reflects a waste of resource for individuals and the organisations who backed them financially or with initial protected time, often resulting in disheartened practitioners and missed opportunities for organisations and the people and communities they serve. Wasted resource is also amplified by the lack of retention of those who do not accept the status quo. Such practitioners and their skills become lost to the organisation that initially supported them. Systems and structures are not consistently working in favour of enabling practitioners to become and remain research active. In some respects and in some, but certainly not all, instances, systems and cultures appears to be resistant to change.

### The ongoing need for enabling infrastructure and systems

The findings of this study echo those of Cromer et al. 17] and Newington et al. [16] by providing personal insights into the lived experience of research activity being de-prioritised in favour of attending to service delivery pressures. As these pressures give no indication of abating in the near future, any thoughts of postponing or deferring action to enable research in practice until demands ease, seem ill-advised. Post-doctoral practitioners are essential to help identify and implement the changes required to reshape and reorient health and social care services to more effectively meet the changing needs of the population. With an aim of system-wide transformation, it is inefficient to leave the creation of viable roles and clinical academic career pathways to individual creativity and tenacity, or to the efforts of forward-thinking leaders and organisations.

Local, context-specific research capacity building programmes and strategies help to ensure congruence with local research priorities [18]. Close alignment of these strategies to wider organisational strategic objectives, business planning, quality strategies and audit activities effectively ‘hard wires’ research, and its supporting infrastructure, as core business [11]. However, the organisational and geographical variability in experience identified by the findings suggests that something more than broad national policy is required to drive consistent and comparable progress in local implementation.

As the findings exemplify, the absence of credible, sustainable, financially viable and equitably accessible career pathways combining research with practice is an ongoing issue for AHPs. It is a long-standing matter that requires urgent attention, not only for AHPs, but for all health and care professions beyond medicine [4–6, 20, 21]. A greater level of direction, new systems, structures and infrastructure, and more effective coordination and the sharing of good practice may help to accelerate and smooth out the rate of progress across the UK. Normalising access to clinical academic career pathways, and normalising an appropriate degree of research engagement for all practitioners, is fundamental to this. What is certain is that repeatedly spotlighting barriers and obstacles, yet failing to take action, will not resolve the issues.

### Harnessing the value added by post-doctoral AHPs

The findings of this study illustrate the personal and professional development accruing from doctoral study for individual AHPs. Beyond the more obvious research-related skills, the value it brings includes the development of analytical and critical thinking skills, practice expertise, time management, resilience, negotiating skills, educational skills, job satisfaction, career development / progression, and enhanced professional standing / credibility. As previously indicated, these areas of growth span all four pillars of practice.

As the findings highlight, in a receptive environment, the value of this personal and professional development has the potential to ripple outwards and positively influence colleagues, services, departments, organisations and even professions – all for the ultimate benefit of the people who access services. Post-doctoral AHPs bring enormous value to organisations but, as we have heard from this study’s participants, they are frequently unrecognised and under-utilised. That in itself generates significant ripple effects, this time in the form of missed opportunities and the associated adverse consequences across the system.

### Limitations

There are of course limitations to this study. The relatively small number of responses, uneven geographical spread across the UK, and the fact that not all AHP disciplines are represented, restricts the opportunity to generalise from the study. Similarly, the convenience and self-selecting nature of the sampling process raises questions about how representative the participants are among AHPs in the UK. However, given the qualitative orientation of this study and its analysis, the aim was to gain a deeper understanding of the significance of participants experiences rather than producing data that is representative and generalizable. It is for others to then assess whether the data presented here, and its interpretation, resonates and is applicable and useful within their own clinical context.

Despite being informed by previous research, the bespoke nature of the questionnaire and lack of formal validation, could mean that questions lacked sensitivity to the complex issues involved in understanding the value of a doctorate for AHPs. However, as noted earlier, the questionnaire was sense checked and adjusted prior to being used in order to minimise any lack of sensitivity. Whilst the qualitative open question approach did not permit clarification or probing of responses, this is true of any qualitative survey. Indeed, Braun et al. [34] demonstrate that online qualitative surveys can deliver rich and nuanced data by promoting a higher level of anonymity than other qualitative approaches and by allowing participants to generate thoughtful (rather than immediate) responses at a time convenient to them.

## Conclusions

This study offers findings that clearly articulate the variability of experiences of post-doctoral AHPs. There are powerful exemplars that role model the optimising of benefits for the individual practitioner, the service, organisation and the community it serves. These provide valuable insights to inspire and inform organisations, services leaders and managers with less experience, helping them to move the research in practice agenda [1–4, 24] forward in their own contexts.

The challenges, barriers and obstacles to post-doctoral research engagement described by participants reflect those that have been acknowledged for many years across a range of health systems and countries (see, for example, 7, 12, 17, 18, 22, 23]. It is important to acknowledge them, but more important is the need to desist from circling around and revisiting them. Instead, the conversation must move forward and generate positive action.

The need to navigate and mitigate the challenges to realise the wide-reaching benefits is fundamental. Reframing perspectives to centre what is to be gained, how it will contribute to enhancing the experiences and outcomes of people accessing services, and what *is* possible, will help to focus attention on how it can be achieved, one incremental step at a time. There is existing evidence identifying approaches that are productive in this regard, once again including some that are well-established (see, for example, 7, 8, 11, 18, 22, 23, 35].

The findings based on the AHP data reported on here demonstrate significant commonalities with our previous findings from nursing and midwifery data [21]. Given the commonality of the broad systems within which these health and care professionals work, this is unsurprising. Notwithstanding the need to address nuanced differences on a more specific basis, it reinforces the need for urgent, system-wide responses to more effectively, consistently and equitably enable career pathways that combine research and practice for what is a very substantial proportion of the health and care workforce in the UK.

### Electronic supplementary material

Below is the link to the electronic supplementary material.

Supplementary Material 1

## Data Availability

The datasets generated and analysed during the current study are not publicly available due to the readily identifiable nature of some participants, given the demographic detail and relative scarcity of some roles mentioned (i.e. there are very few art therapists or music therapists or paramedics with a PhD). Combining this detail with geographical information and job titles could easily identify individuals. However, limited, sufficiently anonymised, data can be made available from the corresponding author on reasonable request.

## Footnotes

1 The umbrella term ‘allied health professions’ encompasses 14 profession groups: art therapy, dietetics, dramatherapy, music therapy, occupational therapy, operating department practice, orthoptics, osteopathy, paramedicine, physiotherapy, podiatry, prosthetics and orthotics, diagnostic and therapeutic radiography and speech and language therapy.

## Abbreviations

ACORN: Addressing Organisational Capacity to do Research Network
AHP: Allied Health Profession/al
CLAHRC: Collaboration for Leadership in Applied Health and Care Research
DIET: Dietician/s
MISC: Art therapist/s, Paramedic/s, Music Therapist/s and Orthoptist/s
NHS: National Health Service
NIHR: National Institute for Health and Care Research
OT: Occupational Therapist/s
POD: Podiatrist/s
PT: Physiotherapist/s
RAD: Radiographer/s
SLT: Speech and Language Therapist/s
UK: United Kingdom
