# Supplementary material for: Understanding the value of a doctorate for allied health professionals in practice in the UK: a survey
Source: BMC Health Serv Res. 2024 May 2;24:566. doi: 10.1186/s12913-024-11035-7 (PMC11064402; doi:10.1186/s12913-024-11035-7)
Supplement: Supplementary file 1 — Supplementary Material 1 [file 12913_2024_11035_MOESM1_ESM.pdf]

How do Nurses, Midwives and Allied Health Professionals (NMAHPs) who are doctoral graduates value their doctoral experience?

Welcome to this online survey. Our study sets out to examine the experience, outcomes and motivation to study for non-medical clinicians who have completed doctorates. This research proposal arose from a Yorkshire and Humber community of practice ACORN (Addressing Capacity in Organisations to do Research Network). This community of practice is funded and facilitated by the NIHR Collaborations for Leadership in Applied Health Research and Care for Yorkshire and Humber (CLAHRC YH) as part of its core principle of research capacity development.

Exploration of NMAHP doctoral experience initially arose because of a collective recognition that pathways to developing an integrated clinical and research career are well established for medical professionals, but comparable opportunities are not well defined for NMAHPs (Cooke et al., 2016a).

The e-survey consists of 3 sections:

Section A: Consent
Section B: Demographic information
Section C: Questions on your doctoral experience

We will use the findings to help support the development of clinical-academic careers for Nurses, Midwives and Allied Health Professionals.

PLEASE NOTE:

This study is not limited to non-medical clinicians who completed their doctorates within the UK.

We therefore welcome completion of this survey by doctorate NMAHPs based around the world.

We recognise that there has been limited research in this area and that our findings will be of interest beyond the UK.

Our information sheet giving more details about the project can be found here : [https://drive.google.com/file/d/1z-DrIO6p51xXiVliesya9mdm7gujdd45/view?usp=sharing]. This will help you decide whether to take part. You are also free to skip questions or stop at any point.

If you would like any further information please contact [redacted] or via our Twitter account @NMAHP_DoctorateStudy

* Indicates required question

Section A: Consent

Please read the following questions carefully and tick the appropriate boxes. Please note these are the only required questions in the whole survey. In sections B and C you are able to skip any questions you would prefer not to answer.

Taking part in the project:

1. I have read and understood the project information sheet dated 4/12/18 (see link). If you answer No to this question please do not proceed with this consent form until you are fully aware of what your participation in the project will mean.) *

   Mark only one oval.

   - Yes
   - No

2. I have been given the opportunity to ask questions about the project *

   Mark only one oval.

   - Yes
   - No

3. I agree to take part in the project. I understand that taking part in the project means completing this e-survey. *

   Mark only one oval.

   - Yes
   - No

4. I understand that my taking part is voluntary and that I can withdraw from the study at any time. I do not have to give any reasons for why I no longer want to take part and there will be no adverse consequences if I choose to withdraw. *

Mark only one oval.

Yes

No

How my information will be used during and after the project

5. I understand that this e-survey is not collecting personal details such as name, phone number and email address *

Mark only one oval.

Yes

No

6. I understand and agree that my words may be quoted in publications, reports web pages and other research outputs. I understand that I will not be named in these outputs unless I specifically request this. *

Mark only one oval.

Yes

No

7. I understand and agree that other authorised researchers will have access to this data only if they agree to preserve the confidentiality of the information requested in this form. *

Mark only one oval.

Yes

No

8. I understand and agree that other authorised researchers may use my data in publications, reports, web pages, and other research outputs, only if they agree to preserve the confidentiality of the information as requested in this form. *

Mark only one oval.

- Yes
- No

9. I agree to assign the copyright I hold in any materials generated as part of this project to The University of Sheffield. *

Mark only one oval.

- Yes
- No

Section B

This first set of questions asks you a little bit about yourself and your doctoral study. Remember, you can skip any questions you would prefer not to answer.

10. 1) What is your current job title?

____

11. 2) What was your job title when you started your doctoral studies?

____

12. 3) In what year did you start your doctoral studies?

____

13. 4) In what year did you graduate?

____

14. 5) In what setting did you undertake your doctoral studies?

Mark only one oval.

- ( ) Inpatient/hospital
- ( ) Community
- ( ) Other: ____________________

15. 6) How was your doctoral study funded? (please tick all that apply)

Tick all that apply.

- [ ] Self-funding
- [ ] University studentship
- [ ] Charitable and Trust funding e.g. Wellcome Trust
- [ ] Employer support
- [ ] UK PhD loans
- [ ] UK National Institute for Health Research studentship
- [ ] UK Research Council studentship
- [ ] Other: ____________________

16. 7) Did you study full-time?

Mark only one oval.

- ( ) Yes
- ( ) No

17. 8) Which of these professional groups do you belong?

Mark only one oval.

- Adult nurses
- Children's nurses
- Mental health nurses
- Midwives
- Health visitors
- Art Therapists
- Drama therapists
- Music therapists
- Chiropodists/podiatrists
- Dietitians
- Occupational therapists
- Operating Department Practitioners
- Orthoptists
- Osteopaths
- Paramedics
- Physiotherapists
- Prosthetists and Orthotists
- Radiographers
- Speech and Language Therapist
- Clinical Scientist
- Pharmacist
- Other: ______________________

The next few questions ask about your location and specifically whereabouts in the UK you are based. If you are non-UK based there is an opportunity to state this and tell us your location.

18. 10) Which region of the UK, are you currently based?

Mark only one oval.

- Non UK
- South East
- London
- North West
- East of England
- West Midlands
- South West
- Yorkshire and the Humber
- East Midlands
- North East
- Scotland
- Wales
- Northern Ireland

19. 11) If you ticked Non UK, where are you based?

____

20. 12) Which region of the UK, were you based when you started your doctoral studies?

Mark only one oval.

- Non UK
- South East
- London
- North West
- East of England
- West Midlands
- South West
- Yorkshire and the Humber
- East Midlands
- North East
- Scotland
- Wales
- Northern Ireland

21. 13) If you ticked Non UK , please tell us where you were based when you started your doctoral studies

____

Value of my doctorate

This section contains several questions which aim to understand what motivated you to study at doctoral level and how you now value that study in terms of personal development, career pathway etc. Remember you can skip any questions you are uncomfortable answering.

22. 14) Why did you decide to study for a doctorate? (please tick all answers that apply)

Tick all that apply.

- [ ] Professional development
- [ ] Change in career
- [ ] Seeking a career in academia
- [ ] Intellectual stimulation and /or curiosity
- [ ] Other: ______________________________

23. 15) If you answered other please explain below

____

____

____

____

____

24. 16) Before starting your doctorate had you taken part in any organised research capacity building activities (e.g. internships, shadowing, short courses etc)

Mark only one oval.

( ) Yes

( ) No

( ) Not sure

25. 17) If you answered yes, could you please briefly outline below

____

____

____

____

____

26. 18) Research suggests that completion of a doctorate has several benefits. To what extent do you agree with the following statements. My doctoral study ...

Mark only one oval per row.

	Strongly agree	Agree	Neither agree or disagree	Disagree	Strongly disagree
**developed my critical thinking skills**	◯	◯	◯	◯	◯
**developed my research and analytical skills**	◯	◯	◯	◯	◯
**deepened my specialist subject knowledge**	◯	◯	◯	◯	◯
**gave me a fresh perspective on my work**	◯	◯	◯	◯	◯
**increased my resilience and confidence**	◯	◯	◯	◯	◯
**made me a more discerning consumer of information**	◯	◯	◯	◯	◯
**extended my networks**	◯	◯	◯	◯	◯
**improved my problem solving skills**	◯	◯	◯	◯	◯
**increased my creativity**	◯	◯	◯	◯	◯

improved my perseverance					
increased my professional credibility					

27. 19) Thinking about your answers above to what extent are you able to utilise these benefits in your current role?

____

____

____

____

____

28. 20) Do you think these skills are valued by your employer

Mark only one oval.

- Yes
- No
- Maybe

29. 21) Please describe how these skills are valued or not by your employer

____

____

____

____

____

30. 22) Research suggests that completion of a doctorate can also be considered a risk. Thinking about your experience to what extent do you agree with the following statements?

Mark only one oval per row.

	Strongly agree	Agree	Neither agree or disagree	Disaggree	Strongly disagree
**My doctoral study was a financial risk**	⬭	⬭	⬭	⬭	⬭
**My post doctoral study earnings are lower than I envisaged**	⬭	⬭	⬭	⬭	⬭
**I am over qualified for my current role**	⬭	⬭	⬭	⬭	⬭
**There are limited opportunities to use skills gained during my doctorate**	⬭	⬭	⬭	⬭	⬭

31. 23) How would you categorise your current role?

Mark only one oval.

- ⬭ Clinicial
- ⬭ Academic
- ⬭ Clinical-academic
- ⬭ Managerial
- ⬭ Teaching
- ⬭ Other: ______________________

32. 24) We would appreciate it if you could tell us a little about your career pathway. In particular, we'd like you to reflect on how you feel your doctorate has (or hasn't) impacted on this career.

33. 25) Please feel free to add any other comments about your doctoral studies

Thank you

Thanks you for completing this survey and for telling us about your doctoral experiences. We will use the findings to support further research capacity building amongst NMAHP.

We would appreciate it if you could forward this survey to your NMAHP colleagues with a doctorate. You can use copy and paste this link to do this
https://goo.gl/forms/TH5SbjZcMf3WWxxG2

This content is neither created nor endorsed by Google.

Google Forms
